# Role of available adjuvant therapies following surgical resection of atypical choroid plexus papilloma—a systematic review and pooled analysis

**DOI:** 10.1093/noajnl/vdaa139

**Published:** 2020-10-25

**Authors:** Amin Tavallaii, Ehsan Keykhosravi, Hamid Rezaee, Camellia Kianbakht

**Affiliations:** 1 Akbar Children Hospital, Neurosurgery Department, Mashhad University of Medical Sciences, Mashhad, Iran; 2 Neurosurgery Department, Mashhad University of Medical Sciences, Mashhad, Iran; 3 Mashhad University of Medical Sciences, Mashhad, Iran

**Keywords:** adjuvant, atypical choroid plexus papilloma, chemoradiation, chemotherapy, radiotherapy

## Abstract

**Background:**

Atypical choroid plexus papilloma is a recently introduced entity with intermediate pathological characteristics. These tumors are relatively rare and the optimal management of these tumors is a matter of debate. Therefore, we performed a systematic review and pooled analysis about the effects of adjuvant therapies on outcome measures of these patients. We also compared these effects on totally and partially resected tumors and pediatric and adult populations.

**Methods:**

A systematic search of 3 databases based on inclusion/exclusion criteria was performed. Data extraction was separately performed by 2 authors, and the summarized data were presented in the form of tables. Pooled estimates of different outcome measures were calculated for each adjuvant therapy and presented separately for studies with pediatric, adult, or mixed populations.

**Results:**

A review of 14 included studies consisting of 144 patients revealed the effect of adjuvant treatment on reduction of tumor recurrence, metastasis, and reoperation rates and increasing survival rates in patients with subtotal tumor resection. This advantage was not seen in the case of gross total tumor resection. Almost all outcome measures were more favorable in the pediatric population.

**Conclusions:**

It can be concluded that whenever gross total resection is not feasible, the implementation of adjuvant therapy can improve the outcome and prognosis. In other cases, it should be decided on an individual basis. Also, more aggressive behavior and higher rates of recurrence and mortality in the adult population suggest the consideration of more aggressive adjuvant treatments for adult patients.

**Key Points**

- Atypical choroid plexus papilloma (CPP) has more aggressive behavior in the adult population compared to the children.
- Adjuvant treatment is recommended following subtotal resection of atypical CPP.
- Adjuvant treatment after total resection should be decided on a case-by-case basis.

**Importance of the Study**

Atypical choroid plexus tumor is a relatively new histopathologic entity that comprises a small percentage of choroid plexus tumors and is considered as an intermediate entity between choroid plexus papilloma and carcinoma. Because of the rarity and recent introduction of these tumors in the literature, there is no consensus about their optimal management following gross total or subtotal surgical resection. Therefore, performing a pooled analysis on the limited available data in the literature can shed a light on this controversial issue in the management of these rare tumors.

Choroid plexus tumors (CPTs) are rare CNS tumors and account for 0.5–0.6% of all intracranial neoplasms in all ages.^1^ These tumors are more common in the pediatric population constituting about 2–4% of all pediatric CNS tumors.^2^ These tumors are most commonly located within the lateral ventricle and the fourth ventricle in children and adults, respectively,^3–5^ although there are reports of these tumors located in unusual locations such as the third ventricle or Luschka foramen.^6^ CPTs were initially classified as WHO grade I choroid plexus papilloma (CPP) and WHO grade III choroid plexus carcinoma (CPC). The presence of any 4 of the malignant characteristics including brisk mitotic activity, nuclear pleomorphism, high cellularity, blurring of the papillary growth pattern, and necrosis leads to the diagnosis of CPC.^7^ The classification of CPTs into CPP and CPC did not cover few tumors with more neoplastic features than can be seen in CPP and not enough neoplastic features to be classified as CPC. This resulted in the coining of the term atypical choroid plexus papilloma (aCPP) as an intermediate entity. After reporting of higher mitotic activity as an atypical feature of CPPs that can affect tumor recurrence rate in a case series,^8^ aCPP was defined as a new entity and classified as grade II by the WHO in 2007.^9^ The histopathologic appearance of aCPP is similar to CPP but with the presence of more than 2 mitoses in the high-power field. Recently, epigenetic characteristics of these tumors such as DNA methylation signature or TP53 somatic mutations are the focus of researchers trying to classify these tumors based on genetic profiles rather than histopathologic characteristics and to present a classification with more prognostic value.^10,11^

The role of adjuvant therapies in the management of CPTs is controversial but the most debate in this regard is about the role of adjuvant therapy in aCPPs which is a relatively newer entity with few cases reported in the literature.^12,13^ For CPP, it is widely accepted that radical surgical removal is the most ideal therapy and no further adjuvant therapy is recommended. Because of the rarity of 2 other subtypes of CPTs, currently implemented therapeutic strategies are based on case reports, and a few larger case series and the optimal management of these tumors are a matter of debate.^3,5,14,15^ Nevertheless, adjuvant therapy in forms of chemotherapy or chemoradiation has a more established role in the management of CPC with reports of a significant increase in overall survival (OS) of affected patients following these treatments.^13,16–18^ In both CPC and aCPP, complete tumor resection seems to significantly improve the prognosis.^19–24^ Whether adjuvant therapies have the same positive effects on outcome measures of aCPP patients as they are reported to have on CPC patients is not demonstrated up to now. We also do not exactly know whether these therapies should be recommended to all aCPP patients or be reserved for subtotally resected aCPP tumors. Therefore, we decided to perform a systematic review and pooled analysis of available data in the literature about the effects of different adjuvant therapies on outcome measures of aCPP patients such as recurrence rate, CSF dissemination, or metastasis and OS or event-free survival (EFS) rates. We also tried to compare these effects on totally and partially resected tumors to provide clues to answer the aforementioned controversies.

## Materials and Methods

### Literature Search and Screening

This systematic review is conducted based on MOOSE reporting guidelines for systematic reviews and meta-analyses of observational studies.^25^ A comprehensive search strategy was planned based on our predefined PICO (Population, Intervention, Comparator, Outcome), including different spellings of “atypical choroid plexus papilloma” AND “Adjuvant” as keywords (Supplementary Material 1). A search of available literature was performed using PubMed, Scopus, and Cochrane library databases. Inclusion criteria were as follows: clinical trials (randomized or non-randomized) and observational studies (cohorts, case series, case reports, and case-control studies) that include participants with a definite histopathologic diagnosis of aCPP (WHO grade II) AND report radiographic outcome or indicate OS or EFS rates. Defined exclusion criteria were as follows: (1) case reports presenting a single case, letters, reviews, conference abstracts, and book chapters, (2) animal and laboratory studies, (3) multiple studies published by the same author on the same population (all are excluded except the most complete and most recently published article), (4) studies without available full text, and (5) studies written in a language other than English.

Upon completion of the database search according to search strategy, titles and abstracts were extracted and authors and journal names were hidden to reduce bias. Two researchers independently and blindly screened extracted abstracts for eligibility according to inclusion and exclusion criteria. Any disagreement was resolved by discussion, and a third researcher was consulted if researchers failed to achieve an agreement. Full texts of eligible studies were retrieved and underwent the same screening process to include eligible full-text articles. Bibliographic data of enrolled articles were checked for relevant articles to be added to the database.

### Data Extraction

The extraction of data was conducted by 2 researchers independently and blindly using a predesigned data extraction form. Data extraction was focused on study characteristics (study type, sample sizes, types of adjuvant treatments, and follow-up durations), demographics of the study population, tumor location, extent of resection, details of outcome measures (radiologic recurrence/progression, remission rates, reoperations, postoperative metastasis/dissemination), survival rates (OS and EFS), conclusions, and suggestions. In case of any critical missing data, the corresponding author(s) were contacted for Supplementary Data. Any discrepancy between 2 researchers was resolved through discussion, and a third researcher was consulted if researchers failed to achieve an agreement.

### Statistical Analysis

Extracted data were inserted into predesigned tables for analysis. Because of the observational nature of available studies in form of cohorts and small case series with available individual patient data in most of the studies, we decided to perform a pooled analysis of available individual patient data by calculating means and rates in studies with pediatric, adult, and mixed populations separately as well as measuring overall pooled estimates for the whole cohort of patients. We also divided patients with available individual data regarding the extent of tumor resection to gross total resection (GTR) and subtotal resection (STR) groups and once more calculated outcome measures and survival rates within these groups to control the influence of this confounding factor on results.

## Results

### Included Studies

**Figure 1.**
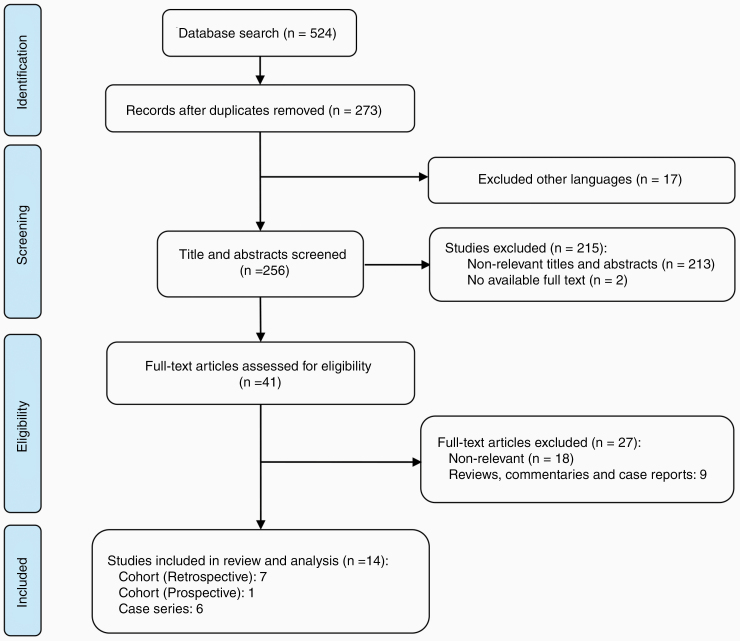
PRISMA chart describing the flow of the article screening procedure.

Search in different databases revealed 273 studies. After excluding non-English articles and screening titles and abstracts, 41 full-text articles were screened for eligibility. Finally, 14 studies were approved for enrollment into review including 7 retrospective cohorts,^13,26–31^ 1 prospective cohort,^15^ and 6 case series^14,32–36^ (Figure 1). A summary of the included studies is presented in Table 1.

**Table 1.** Summary of the Included Studies and Demographic Data of Included Patients Separately for Studies With Pediatric, Adult, and Mixed Populations (numbers in the parentheses present ranges and percentages)

	Study	Study Design	Time Period	*N*	Study Groups (*n*)	Mean Age (years)	Male *n* (%)	Female *n* (%)
*Pediatric*	Wrede et al., 2009^15^	Cohort (prospective)	2000–2008	24	No adjuvant (GTR) (15)	0.6 (0–9)	12 (50%)	12 (50%)
					Chemotherapy (STR) (5)			
					Chemoradiation (STR above 3) (4)			
	Serowka et al., 2010^27^	Cohort (retrospective)	1979–2005	2	No adjuvant (GTR) (1)	0.3 (0.2–0.3)	2 (100)	0
					Chemotherapy (STR) (1)			
	Lam et al., 2013^33^	Case series	1973–2008	12	No adjuvant (12)	2.1 (0–6)	5 (42%)	7 (58%)
	Koh et al., 2014^14^	Case series	1993–2012	7	No adjuvant (7)	0.6	4 (58%)	3 (42%)
	Passariello et al., 2015^13^	Cohort (retrospective)	2000–2014	4	No adjuvant (GTR) (2)	5 (0.6–16)	1 (25%)	3 (75%)
					Chemotherapy (STR) (2)			
	Siegfried et al., 2017^29^	Cohort (retrospective)	2000–2012	26	No adjuvant (GTR:14/STR:6) (20)	1.9 (0–12)	14 (54%)	12 (46%)
					Chemotherapy (GTR:3, STR:1) (4)			
					Chemoradiation (GTR:1/STR:1) (2)			
	Zhou et al., 2018^30^	Cohort (retrospective)	2011–2016	17	No adjuvant (GTR) (5)	4.2 (0.6–14)	12 (70%)	5 (30%)
					Chemotherapy (GTR) (10)			
					Radiotherapy (STR) (1)			
					Chemoradiation (STR) (1)			
	Dash et al., 2019^36^	Case series	2010–2018	2	No adjuvant (GTR) (2)	0.2 (0.1–0.3)	2 (100%)	0
	Hosmann et al., 2019^31^	Cohort (retrospective)	1991–2016	5	No adjuvant (GTR) (5)	0.7 (0.2–1.3)	2 (40%)	3 (60%)
*Pooled estimates*			1973–2018	99		1.9 (0–16)	54 (54.5%)	45 (45.5%)
*Adult*	Bostrom et al., 2011^32^	Case series	1990–2008	2	No adjuvant (2)	37.5 (20–55)	1 (50%)	1 (50%)
	Turkoglu et al., 2014^28^	Cohort (retrospective)	2002–2012	4	No adjuvant (GTR) (2)	—	—	—
					Radiotherapy (STR) (2)			
	Hosmann et al., 2019^31^	Cohort (retrospective)	1991–2016	6	No adjuvant (GTR:2/STR:1) (3)	49.6 (34–71)	4 (67%)	2 (33%)
					Chemotherapy (STR) (1)			
					Radiotherapy (GTR) (2)			
*Pooled estimates*			1990–2016	12		46.5 (20–71)	5 (62.5%)	3 (37.5%)
*Mix*	Menon et al., 2010^26^	Cohort (retrospective)	1998–2009	4	No adjuvant (3)	22.1 (0.7–43)	3 (75%)	1 (25%)
					Chemotherapy (1)			
	Bohara et al., 2015^34^	Case series	1995–2014	3	No adjuvant (GTR) (2)	24.1 (0.5–46)	0	3 (100%)
					Chemoradiation (STR) (1)			
	Cannon et al., 2015^35^	Case series	2004–2009	26	No adjuvant (26)	22.8 (0–55)	11 (42%)	15 (58%)
*Pooled estimates*			1995–2014	33		22.8 (0–55)	14 (42%)	19 (58%)
*Total estimates*			1973–2018	144		9.3 (0–71)	73 (52%)	67 (48%)

GTR, gross total resection; STR, subtotal resection.

### Demographic Data

There was a total of 144 patients with aCPP including 107 patients who had received no adjuvant treatment, 24 patients who had been treated with chemotherapy postoperatively, and 8 and 5 patients who had undergone chemoradiation and radiotherapy, respectively. Eight of the studies included pediatric patients,^13–15,27,29,30,33,36^ and only 2 studies totally consisted of the adult patient population.^28,32^ Also, 4 studies had a mixed pediatric and adult population^26,31,34,35^ with only one of them providing individual patient data for separate data extraction for the pediatric and adult populations^31^ (Table 1). To prevent readers from getting confused by these heterogeneous types of studies with different included populations, we developed a strategy to demonstrate the results of these populations separately within tables. Of course, we also calculated the pooled estimates for the total included patient population. Ages of patients ranged from 0–16 years (mean: 1.9), 20–71 years (mean: 46.5), and 0–55 years (mean: 22.8) for pediatric, adult, and mixed populations, respectively. Overall, no gender predilection was seen in all included patients (M/F: 1.08) although, in the adult population, the M/F ratio of 1.66 was calculated (Table 1).

### Preoperative Imaging Findings

Interestingly, the most common tumor location in both pediatric and adult populations was the lateral ventricle, followed by the third ventricle and fourth ventricle in decreasing order but, in the mixed population, tumor location within the fourth ventricle was slightly more common than the lateral ventricle. Overall, in all included patients, lateral ventricle was the most common location for aCPP followed by third and fourth ventricles. Radiologic evidence of preoperative dissemination or metastasis was only seen in 5% of the pediatric population which was equivalent to 3% of total included patients with aCPP (Table 2).

**Table 2.** Summary of Preoperative Imaging Findings, the Extent of Tumor Resection, and Follow-up Duration of Included Studies Separately for Studies With Pediatric, Adult, and Mixed Populations (numbers in the parentheses present ranges and percentages)

	Study	Tumor Location			Preoperative Metastasis	Extent of Resection		Mean Follow-up Duration (m)				
		Lateral Ventricle	Fourth Ventricle	Third Ventricle		GTR	STR	Overall	No Adjuvant	Chemotherapy	Radiotherapy	Chemoradiation
*Pediatric*	Wrede et al., 2009^15^	20 (83%)	1 (4%)	3 (12%)	5 (20%)	15 (62%)	9 (38%)	30 (20–39)	20	39	—	39
	Serowka et al., 2010^27^	1 (50%)	0	1 (50%)	0	1 (50%)	1 (50%)	2 (1–3)	1	3	—	—
	Lam et al., 2013^33^	—	—	—	0	8 (67%)	4 (33%)	38 (21–56)	38 (21–56)	—	—	—
	Koh et al., 2014^14^	6 (86%)	0	1 (14%)	0	7 (100%)	0	69	69	—	—	—
	Passariello et al., 2015^13^	4 (100%)	0	0	0	2 (50%)	2 (50%)	90.7 (20–144)	49.5 (20–79)	114	—	—
	Siegfried et al., 2017^29^	22 (85%)	0	4 (15%)	0	18 (69%)	8 (31%)	—	—	—	—	—
	Zhou et al., 2018^30^	12 (70%)	4 (24%)	1 (6%)	0	15 (88%)	2 (12%)	12 (2–53)	—	—	—	—
	Dash et al., 2019^36^	1 (50%)	0	1 (50%)	0	2 (100%)	0	3	3	—	—	—
	Hosmann et al., 2019^31^	3 (60%)	0	2 (40%)	0	5 (100%)	0	61.4 (4–182)	61.4 (4–182)	—	—	—
*Pooled estimates*		69 (79%)	5 (6%)	13 (15%)	5 (5%)	73 (74%)	26 (26%)	34.8 (1–182)	22.3 (1–182)	53.2 (3–114)	—	39
*Adult*	Bostrom et al., 2011^32^	1 (50%)	0	1 (50%)	0	2 (100%)	0	51 (28–74)	51 (28–74)	—	—	—
	Turkoglu et al., 2014^28^	—	—	—	0	2 (50%)	2 (50%)	68.4 (48–84)	68.4 (48–84)	—	68.4 (48–84)	—
	Hosmann et al., 2019^31^	6 (100%)	0	0	0	4 (67%)	2 (33%)	97 (3–190)	137.2 (108–190)	119	51.6 (3–49)	—
*Pooled estimates*		7 (87%)	0	1 (13%)	0	8 (67%)	4 (33%)	79.8 (3–190)	93 (28–190)	119	60 (3–84)	—
*Mix*	Menon et al., 2010^26^	2 (50%)	2 (50%)	0	0	2 (50%)	2 (50%)	26 (18–44)	26 (18–44)	24	—	—
	Bohara et al., 2015^34^	1 (33%)	2 (67%)	0	0	2 (67%)	1 (33%)	96 (52–171)	111 (52–171)	—	—	66
	Cannon et al., 2015^35^	—	—	—	0	15 (58%)	11 (42%)	—	—	—	—	—
*Pooled estimates*		3 (43%)	4 (57%)	0	0	19 (57%)	14 (43%)	56 (18–171)	62.4 (18–171)	24	—	66
*Total estimates*		79 (77%)	9 (9%)	14 (14%)	5 (3%)	100 (69%)	44 (31%)	40.5 (1–190)	34.7 (1–190)	56.8 (3–119)	60 (3–84)	44.4 (39–66)

GTR, gross total resection; STR, subtotal resection.

### The Extent of Resection

In the pediatric population, GTR was achieved in 74% of patients but aCPP tumors in adult and mixed populations were less amenable to GTR with 67% and 57% GTR rates, respectively. Also, in all 144 patients, GTR was feasible in 69% of cases (Table 2).

### Adjuvant Treatment Protocols

Among the 14 included studies, the overall chemotherapy regimen or radiotherapy protocol had been indicated in only 4 studies.^15,29,31,34^ Even in these 4 studies, the significant details of the adjuvant treatment protocol were missing. The most used chemotherapeutic agents were Etoposide, Vincristine, Cisplatine, Cyclophosphamide, or Ifosfomide but other agents such as Carboplatine or Thalidomide were also mentioned. No dosing data was available for these agents. The most commonly used radiotherapy protocol was local brain radiation with 54 Gy (30 fractions of 1.8 Gy) for non-metastatic tumors and craniospinal irradiation with 35.2 Gy (22 fractions of 1.6 Gy) and a local brain boost up to a total of 54 Gy for metastatic tumors. Unfortunately, this detailed data were only available in 3 of the included studies, 1 of which included the pediatric patients and the 2 other studies consisted of mixed adult and pediatric patients.^15,31,34^

### Radiologic Outcome

Pooled estimates of mean follow-up duration for all included patients were 34.7 (1–190), 56.8 (3–119), 60 (3–84), and 44.4 (39–66) months for no adjuvant, chemotherapy, radiotherapy, and chemoradiation cohorts, respectively, with a calculated overall estimate of 40.5 months for the whole review cohort. Details of follow-up durations in each population are separately demonstrated in Table 2. An average of 8% of patients who had received no adjuvant therapy had shown signs of tumor progression or recurrence in the follow-up period. Interestingly, these rates were 33%, 25%, and 20% for patients who had been treated with chemotherapy, radiotherapy, or chemoradiation as an adjuvant, respectively (Table 3). These conflicting results seemed to be due to the potential confounding effect of the extent of resection which is comprehensively discussed in the following sections. The recurrence rate was considerably higher in the adult population compared to pediatric patients in almost all treatment arms. Overall mean time to recurrence was 35 months with longer times for patients who had received no adjuvant treatment (31 months) compared to patients who had been treated with various adjuvant treatments. Similar to recurrence rates, these findings seem to be confounded by the extent of tumor resection. In the pediatric population, tumor dissemination/metastasis has occurred in one patient with a subtotally resected tumor despite postoperative chemotherapy. Similarly, one of the adult patients has experienced postoperative dissemination/metastasis following subtotal tumor resection without any further adjuvant treatment. Generally, 14% of patients in the no adjuvant cohort had been undergone reoperation in the follow-up period while the pooled estimates of reoperation rates were 33% and 20% for chemotherapy and chemoradiation cohorts. The overall estimated rates of complete remission for no adjuvant, chemotherapy, and chemoradiation cohorts were 76%, 65%, and 71%, respectively. All these data are presented in full detail for each cohort and each patient population in Table 3.

**Table 3.** Summary of Postoperative Outcome Measures Following Each Adjuvant Therapy Calculated Separately for Studies With Pediatric, Adult, and Mixed Populations

	Study	Recurrence/Progression					Mean Time to Recurrence (m)					Postoperative Metastasis					Reoperation					Radiologic Outcome							
		Overall	No adjuvant	Chemotherapy	Radiotherapy	Chemoradiation	Overall	No adjuvant	Chemotherapy	Radiotherapy	Chemoradiation	Overall	No adjuvant	Chemotherapy	Radiotherapy	Chemoradiation	Overall	No adjuvant	Chemotherapy	Radiotherapy	Chemoradiation	No adjuvant		Chemotherapy		Radiotherapy		Chemoradiation	
																						CR	PR	CR	PR	CR	PR	CR	PR
*Pediatric*	Wrede et al., 2009^15^	2	1	0	—	1	—	25	—	—	27	—	—	—	—	—	1	0	0	—	1	—	—	20%	80%	—	—	50%	50%
	Serowka et al., 2010^27^	—	—	—	—	—	—	—	—	—	—	—	—	—	—	—	—	—	—	—	—	—	—	—	—	—	—	—	—
	Lam et al., 2013^33^	0	0	—	—	—	—	—	—	—	—	0	0	—	—	—	—	—	—	—	—	—	—	—	—	—	—	—	—
	Koh et al., 2014^14^	0	0	—	—	—	—	—	—	—	—	0	0	—	—	—	0	0	—	—	—	—	—	—	—	—	—	—	—
	Passariello et al., 2015^13^	2	0	2	—	—	10	—	10	—	—	1	0	1	—	—	2	0	2	—	—	—	—	—	—	—	—	—	—
	Siegfried et al., 2017^29^	9	—	—	—	—	—	—	—	—	—	—	—	—	—	—	8	—	—	—	—	90%	10%	100%	0	—	—	100%	0
	Zhou et al., 2018^30^	8	—	—	—	—	—	—	—	—	—	0	0	0	0	0	—	—	—	—	—	60%	40%	80%	20%	0	100%	100%	0
	Dash et al., 2019^36^	0	0	—	—	—	—	—	—	—	—	0	0	—	—	—	0	0	—	—	—	—	—	—	—	—	—	—	—
	Hosmann et al., 2019^31^	1	1	—	—	—	—	—	—	—	—	0	0	—	—	—	1	1	—	—	—	80%	20%	—	—	—	—	—	—
*Pooled estimates*		23%	5%	28%	—	25%	10	25	10	—	27	2%	0	8%	0	0	18%	3%	28%	—	25%	83%	17%	68%	32%	0	100%	71%	29%
*Adult*	Bostrom et al., 2011^32^	0	0	—	—	—	—	—	—	—	—	0	0	—	—	—	0	0	—	—	—	—	—	—	—	—	—	—	—
	Turkoglu et al., 2014^28^	0	0	—	0	—	—	—	—	—	—	0	0	—	0	—	0	0	—	0	—	—	—	—	—	—	—	—	—
	Hosmann et al., 2019^31^	5	3	1	1	—	—	—	—	—	—	1	1	0	0	—	4	3	1	0	—	0	33%	0	100%	0	100%	—	—
*Pooled estimates*		42%	43%	100%	25%	—	—	—	—	—	—	8%	14%	0	0	—	33%	43%	100%	0	—	0	33%	0	100%	0	100%	—	——
*Mix*	Menon et al., 2010^26^	2	2	0	—	—	60	60	—	—	—	2	2	0	—	—	2	2	0	—	—	—	—	—	—	—	—	—	—
	Bohara et al., 2015^34^	0	0	—	—	0	—	—	—	—	—	0	0	—	—	0	0	0	—	—	0	—	—	—	—	—	—	—	—
	Cannon et al., 2015^35^	—	—	—	—	—	—	—	—	—	—	1	1	—	—	—	—	—	—	—	—	—	—	—	—	—	—	—	—
*Pooled estimates*		28%	40%	0	—	0	60	60	—	—	—	9%	10%	0	—	0	28%	40%	0	—	0	—	—	—	—	—	—	—	—
*Total estimates*		25%	8%	33%	25%	20%	35	31	10	—	27	5%	6%	7%	0	0	21%	14%	33%	0	20%	76%	18%	65%	35%	0	100%	71%	29%

CR, complete remission; PR, partial remission.

### Survival Rates

Different measures were used by included studies to report the survival rates including 2-year OS, 5-year OS, 2-year EFS, and 5-year EFS. In order to be able to include most of these measures in the pooled analysis, we selected the 2-year OS and EFS as our measures of interest. In cases in which 5-year survival rates were reported to be 100%, this rate was also considered for a 2-year survival rate. As a result of this strategy, in the pediatric population, a 2-year OS of 93% was calculated for no adjuvant group while the patients who had received adjuvant therapies had shown a 2-year OS of 100%. This was also seen in the adult population with 2-year OS rates of 71% and 100% for no adjuvant and chemotherapy/radiotherapy groups, respectively. A complete array of calculated pooled estimates for OS, EFS, and mortality rates of each treatment strategy in each age group is provided in Table 4.

**Table 4.** Summary of Survival Rates Following Each Adjuvant Therapy Calculated Separately for Studies With Pediatric, Adult, and Mixed Populations (numbers in the parentheses present percentages)

	Study	Overall Survival					Event-Free Survival					Mortality				
		Overall	No adjuvant	Chemotherapy	Radiotherapy	Chemoradiation	Overall	No adjuvant	Chemotherapy	Radiotherapy	Chemoradiation	Overall	No adjuvant	Chemotherapy	Radiotherapy	Chemoradiation
*Pediatric*	Wrede et al., 2009^15^	89%	—	—	—	—	83%	—	—	—	—	1	0	0	—	1
	Serowka et al., 2010^27^	—	—	—	—	—	—	—	—	—	—	1	0	1	—	—
	Lam et al., 2013^33^	83%	83%	—	—	—	—	—	—	—	—	2	2	—	—	—
	Koh et al., 2014^14^	100%	100%	—	—	—	100%	100%	—	—	—	0	0	—	—	—
	Passariello et al., 2015^13^	100%	100%	100%	—	—	50%	100%	—	—	—	0	0	0	—	—
	Siegfried et al., 2017^29^	—	—	—	—	—	—	—	—	—	—	1	1	0	—	0
	Zhou et al., 2018^30^	100%	100%	100%	100%	100%	48%	—	—	—	—	0	0	0	0	0
	Dash et al., 2019^36^	—	—	—	—	—	—	—	—	—	—	1	1	—	—	—
	Hosmann et al., 2019^31^	100%	100%	—	—	—	80%	80%	—	—	—	0	0	—	—	—
*Pooled estimates*		93%	93%	100%	100%	100%	72%	93%	—	—	—	6 (6%)	4 (6%)	1 (4%)	0	1 (14%)
*Adult*	Bostrom et al., 2011^32^	100%	100%	—	—	—	100%	100%	—	—	—	0	0	—	—	—
	Turkoglu et al., 2014^28^	100%	100%	—	100%	—	100%	100%	—	100%	—	0	0	—	0	—
	Hosmann et al., 2019^31^	67%	33%	100%	100%	—	33%	0	—	50%	—	2	2	0	0	—
*Pooled estimates*		83%	71%	100%	100%	—	66%	57%	—	75%	—	2 (17%)	2 (28%)	0	0	—
*Mix*	Menon et al., 2010^26^	100%	100%	100%	—	—	50%	33%	100%	—	—	0	0	0	—	—
	Bohara et al., 2015^34^	100%	100%	—	—	100%	100%	100%	—	—	100%	0	0	—	—	0
	Cannon et al., 2015^35^	—	—	—	—	—	—	—	—	—	—	3	3	—	—	—
*Pooled estimates*		100%	100%	100%	—	100%	71%	60%	100%	—	100%	3 (9%)	3 (10%)	0	—	0
*Total estimates*		92%	90%	100%	100%	100%	71%	77%	100%	75%	100%	8%	6%	4%	0	12%

### Outcome Measures Considering the Extent of Resection

**Figure 2.**
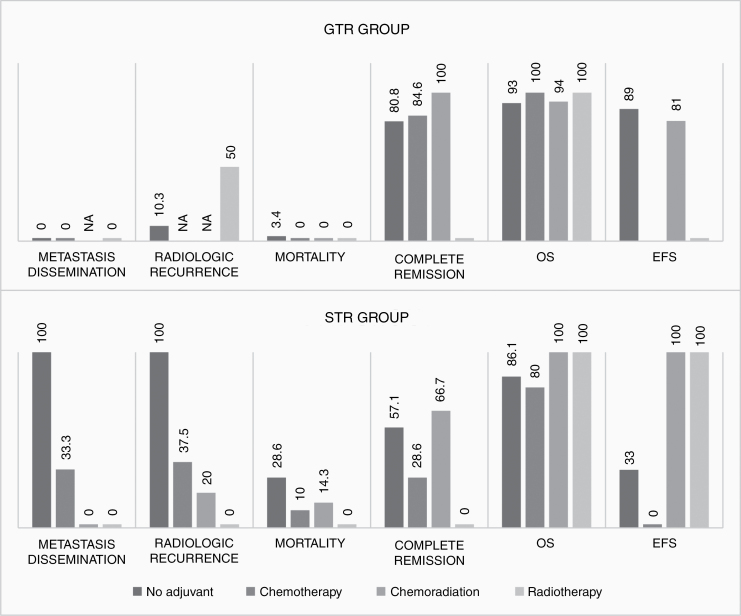
Bar charts demonstrating pooled estimates of various outcome measures following each adjuvant therapy separately calculated for GTR and STR groups, respectively.

Most of the included studies had followed the strategy of implementing no adjuvant treatment for patients with gross total removal of the tumor and reserving chemotherapy, chemoradiation, or radiotherapy for patients with subtotal tumor resection. Only in 3 studies, initiation of adjuvant treatment was not based on this strategy and it was decided on an individual basis^29–31^ (Table 1). As can be seen, this can be a potential confounding factor in the comparison of outcome measures between patients who had received no adjuvant treatment and those had been treated with various adjuvant therapies. So, we divided the patients with separately available outcome data for various adjuvant therapies to GTR and STR groups and calculated the outcome measures for these groups separately (Supplementary Material 2). In both GTR and STR groups, the mortality rate was lower in all patients who had received adjuvant therapies compared to the patients without any adjuvant treatment. Similarly, adjuvant therapies also resulted in a higher OS rate in both STR and GTR groups. Although the pooled estimate of OS for patients who had received chemotherapy in the STR group was slightly lower compared to patients who had not received any adjuvant therapies. An almost similar pattern was seen in pooled estimates of EFS. Another interesting finding was the considerable decrease in estimates of radiologic recurrence and rate of postoperative tumor dissemination/metastasis following adjuvant therapies in the STR group while there was not such an effect observed in the GTR group that is a clear approval of the presumed role for the extent of resection as a confounding factor (Figure 2).

## Discussion

 The role of adjuvant therapies in the management of CPTs is controversial but the most debate in this regard is about the role of adjuvant therapy in aCPPs which is a relatively newer entity with fewer cases reported in the literature.^12,13^ Because of the rarity of aCPPs, currently implemented therapeutic strategies are not based on high-level evidence and the optimal management of these tumors is still a matter of debate.^3,5,14,15^ While it seems that complete tumor resection significantly improves the prognosis, it is not clearly demonstrated whether the adjuvant therapies have the same positive effects on outcome measures of aCPP patients as they are reported to have on CPC patients.^19–24^ It is also controversial whether these adjuvant therapies should be recommended to all aCPP patients or be reserved for subtotally resected aCPP tumors. In our review, we tried to answer these questions by pooling the available data in the literature and presenting a higher level of evidence.

### Preoperative and Operative Parameters

As we know, CPTs, in general, are more commonly seen in the supratentorial and infratentorial regions in the pediatric and adult populations, respectively.^3–5^ As an interesting finding of this review, the most common location of aCPP was the lateral ventricle followed by third and fourth ventricles in both pediatric and adult populations. This can be an important finding from an epidemiologic and diagnostic point of view.

Preoperative metastasis is not a rare finding in CPC but its incidence in aCPP patients is unknown. In this review, only one study reported 5 out of 24 patients to have preoperative metastasis, and this unusually high rate of metastasis compared to other included studies may be a result of referral or selection bias and should be considered as an outlier.^15^

GTR of CPPs is almost always feasible given the fact that these tumors do not invade surrounding brain parenchyma. On the contrary, invasion to the adjacent tissue is common in CPCs and these tumors are less amenable to GTR. As can be seen in this review, the GTR rate of aCPPs falls somewhere in between with an overall GTR rate of 69% for the whole review cohort. It is also evident that the GTR/STR ratio is lower in the adult population compared to pediatric patients.

### Outcome Measures

In this review, the mean follow-up duration of patients who had received adjuvant therapies was higher than patients without any adjuvant treatments but even in these patients with no adjuvant treatment, the mean follow-up duration was long enough (34.7 months) to ensure a reliable estimate of outcome measures.

In the whole review cohort, an 8% recurrence rate was calculated for patients who had not received any adjuvant treatment. However, after dividing the patients into 2 GTR and STR groups based on the extent of resection, all patients in the STR group had shown recurrence without receiving any adjuvant treatment while this rate was considerably lower (10%) in the GTR group. This underlines the extent of resection as one of the most important prognostic factors of patients with aCPPs similar to other grades of CPTs.

In this review, as the result of a larger sample size compared to available case series and cohorts in the literature, we were able to divide the review cohort into GTR and STR groups to control the confounding effect of the extent of resection on outcome measures. As can be seen in primary results, the adjuvant cohort paradoxically demonstrates higher rates of recurrence/progression, reoperation, and partial remission compared to no adjuvant cohort but, interestingly after controlling for the extent of resection as a potential confounding factor, the considerable effect of adjuvant treatment in the reduction of recurrence/progression, metastasis/dissemination, and reoperation rates is evident. This is also true for survival rates with a clear survival advantage (both OS and EFS) of the adjuvant cohort over the no adjuvant cohort.

Even in the GTR group, there were few positive effects on outcome measures (although brief) observed for adjuvant therapy. For example in patients who had received adjuvant treatments (except radiotherapy) rate of complete remission was higher especially for chemoradiation. Also, patients who had received adjuvant treatments (despite GTR) had shown lower mortality rates and higher OS. In the GTR group, only 2 patients had received radiotherapy alone as the adjuvant treatment, which is a statistically small sample size and the inconsistency of results for these 2 patients with other results of the adjuvant cohort is related to this small sample size and can be considered as an outlier.

### Epigenetic Profile

There are recent interesting reports available in the literature that show the correlation between the epigenetic profile of the CPTs and patient outcomes. In an attempt to present an epigenetic-based classification of CPTs with prognostic value, Thomas et al.^11^ used the DNA methylation hierarchical clustering technique which resulted in 3 clinically distinct subgroups. Methylation clusters 1 and 2 have consisted of CPP and aCPP patients of the pediatric and adults populations, respectively. Whereas methylation cluster 3 consisted of all 3 histological subgroups of the CPTs (CPP, aCPP, and CPC) in the pediatric population. Patients in cluster 3 had significantly lower OS and higher tumor progression. Interestingly, the prognosis and outcome of patients with aCPP can be classified as low risk (clusters 1 and 2) and high risk (cluster 3). TP53 mutation status was also introduced as a prognostic factor. All TP53 mutations had been seen in patients categorized as cluster 3 and no patient in cluster 1 or 2 had shown TP53 mutation. Unfortunately, the epigenetic profile was not addressed in any of the included studies, and we were not able to investigate these findings in our review.

### Pediatric vs Adult Population

This is the first review that provides the opportunity to compare outcome measures of aCPP between pediatric and adult populations. This separation of adult and pediatric population seems more important when we consider the differences in responses to and consequences of various adjuvant therapies between these 2 physiologically different populations. Interestingly, it seems that tumor recurrence/progression, postoperative metastasis/dissemination, and reoperation rate is generally higher in the adult population regardless of implemented adjuvant treatment strategy. Also, adult patients with aCPP seem to have a lower complete remission rate, the extent of resection, OS, and EFS compared to the pediatric population. These findings underline the more aggressive behavior and less favorable outcome of adult patients. Therefore, older age can be suggested as a negative prognostic factor for aCPP.

### Strengths and Limitations

 This is the first review dedicated specifically to the role of adjuvant treatment in the less-studied aCPP entity. We tried to perform a comprehensive and reliable review of the literature and to avoid errors or biases by involving 2 authors in critical steps such as article screening and data extraction. We also prepared this review according to standardized MOOSE reporting guidelines to augment its quality.^25^ Fortunately, the mean follow-up duration in all groups was considerably long enough which ensures reliable estimates of long-term outcomes. Another strength of our review is that we divided the review cohort into 2 separate age-based cohorts of adults and pediatric patients, which resulted in the ability to compare the tumor characteristics and outcome measures between these 2 physiologically different populations. Certainly, we faced some limitations during the review process. Like many other entities of neurosurgery, almost all of the included studies are case series and retrospective observational studies with small sample sizes that impede the achievement of more reliable conclusions. Among 144 patients present in the whole review cohort, 107 patients had not received any adjuvant treatments while various adjuvant treatments had been implemented for the other 37 patients. This asymmetry in sample sizes of these 2 arms of our review is a limitation that is inevitable in the pooled analysis of case series and retrospective cohorts and a large size prospective study is needed to address this shortcoming.

## Conclusions

 In this review, we have shown that the nature and behavior of aCPP are not the same in all patients with more aggressive behavior and higher rates of recurrence and mortality in the adult population compared to pediatric patients, which recommend consideration of more aggressive adjuvant treatments for adult patients. We also demonstrated a clear advantage of adjuvant treatment in patients who had undergone STR in terms of decreasing the recurrence rate, metastasis, reoperation rate, and increasing the OS and EFS. Despite the small benefits of adjuvant treatments in patients who had undergone GTR, it can not be recommended to all these patients and it should be decided on an individual basis. The optimal adjuvant treatment for aCPP patients can not be concluded from this review, and there is a significant gap in the literature about this entity which can be addressed by conducting a long-term prospective cohort with multiple treatment arms.

## Compliance With Ethical Standards

*Human participant or animal involvement*. This is a systematic review of available data and there is no human participant or animal directly or indirectly involved in this study.

*Informed consent or ethical approval.* Because systematic reviews do not present individual data that can disclose patients’ identity, informed consent acquisition is not applicable. On the other hand, because systematic reviews use publicly available data for interpretation of results and do not include any interventions, institutional ethics approval was not obtained. However, all relevant aspects of ethics in the research were considered and followed throughout the study.

## Supplementary Material

vdaa139_suppl_Supplementary_Files_1

vdaa139_suppl_Supplementary_Files_2

## Notes

**Authorship statement**. Conception and design: A.T.; acquisition of data: A.T., E.K., and C.K.; analysis and interpretation of data: A.T. and H.R.; drafting the article: A.T. and E.K; critically revising the article: A.T., H.R., and C.K; final approval: A.T., E.K., H.R., and C.K.
