# Supplementary material for: Role of available adjuvant therapies following surgical resection of atypical choroid plexus papilloma—a systematic review and pooled analysis
Source: Neurooncol Adv. 2020 Oct 25;2(1):vdaa139. doi: 10.1093/noajnl/vdaa139 (PMC7712806; doi:10.1093/noajnl/vdaa139)
Supplement: vdaa139_suppl_Supplementary_Files_1 [file vdaa139_suppl_supplementary_files_1.docx]

**Population**

- Atypical choroid plexus papilloma
- Choroid plexus papilloma grade II

“Atypical choroid plexus papilloma” OR “Choroid plexus papilloma grade II” OR “Choroid plexus papilloma grade-II” OR “Choroid plexus papilloma, grade II” OR “Choroid plexus papilloma, grade-II”

**AND**

**Intervention**

- Adjuvant
- Chemotherapy
- Radiotherapy
- Chemoradiotherapy
- Chemoradiation
- Radiation

“Adjuvant” OR “Chemotherapy” OR “Radiotherapy” OR “Radiotherapy” OR “Chemoradiotherapy” OR “Chemoradiation” OR “Radiation”

**AND**

**Comparator**

- Surgery
- Resection
- Surgical management

“Surgery” OR “Resection” OR “Surgical management”

**AND**

**Outcome**

- Survival
- Recurrence
- Complication
- Cure

“Survival” OR “Recurrence” OR “Complication” OR “Cure”

**Study type**

- RCT
- Non-randomized controlled trial
- Cohort
- Case control
- Case series
- Cross sectional
