# Supplementary material for: Role of available adjuvant therapies following surgical resection of atypical choroid plexus papilloma—a systematic review and pooled analysis
Source: Neurooncol Adv. 2020 Oct 25;2(1):vdaa139. doi: 10.1093/noajnl/vdaa139 (PMC7712806; doi:10.1093/noajnl/vdaa139)
Supplement: vdaa139_suppl_Supplementary_Files_2 [file vdaa139_suppl_supplementary_files_2.docx]

|  | | **Metastasis/ Dissemination** | **Radiologic Recurrence** | **Complete Remission** | **Mortality** | **Overall Survival** | **Event-Free Survival** |
| --- | --- | --- | --- | --- | --- | --- | --- |
| **GTR** | No adjuvant | 0 | 10.3% | 80.8% | 3.4% | 93% | 89% |
|  | Chemotherapy | 0 | --- | 84.6% | 0 | 100% | --- |
|  | Chemoradiation | --- | --- | 100% | 0 | 94% | 81% |
|  | Radiotherapy | 0 | 50% | 0 | 0 | 100% | 0 |
| **STR** | No adjuvant | 100% | 100% | 57.1% | 28.6% | 86.1% | 33% |
|  | Chemotherapy | 33.3% | 37.5% | 28.6% | 10% | 80% | 0 |
|  | Chemoradiation | 0 | 20% | 66.7% | 14.3% | 100% | 100% |
|  | Radiotherapy | 0 | 0 | 0 | 0 | 100% | 100% |

**Supplementary material 2** Summary of outcome measures following each adjuvant therapy calculated separately for patients who had undergone gross total or subtotal resection of the tumor. GTR: gross total resection, STR: subtotal resection

Adjuvant treatment

Outcome measures
